# Vision-Based Vehicle State and Behavior Analysis for Aircraft Stand Safety

**DOI:** 10.3390/s26061821

**Published:** 2026-03-13

**Authors:** Ke Tang, Liang Zeng, Tianxiong Zhang, Di Zhu, Wenjie Liu, Xinping Zhu

**Affiliations:** 1 College of Air Traffic Management, Civil Aviation Flight University of China, Guanghan 618307, China; tangke@cafuc.edu.cn (K.T.); zengliang@cafuc.edu.cn (L.Z.); zw749846@gmail.com (D.Z.); lwjwzq1114@outlook.com (W.L.); 2 Institute of Software and Multimedia Technology, Technische Universität Dresden, 01187 Dresden, Germany; tianxiong.zhang@tu-dresden.de

**Keywords:** aircraft stand safety, monocular visual localization, semantic map, spatiotemporal behavior analysis, vehicle violation detection in stands, lightweight object detection, airport ground safety

## Abstract

With the continuous elevation of aviation safety standards, accurate monitoring of ground support vehicles in aircraft stand areas has become a critical task for enhancing overall aircraft stand operational safety. Given the limitations of existing surface movement radar and multi-camera surveillance systems in terms of cost, deployment complexity, and coverage, this paper proposes a lightweight vision-based framework for vehicle state perception and spatiotemporal behavior analysis oriented toward aircraft stand safety. Leveraging existing fixed monocular monitoring resources in the stand area, the framework first establishes a precise mapping from image pixel coordinates to the physical plane through self-calibration and homography transformation utilizing scene line features, thereby achieving unified spatial measurement of vehicle targets. Subsequently, it integrates an improved lightweight YOLO detector (incorporating Ghost modules and CBAM for noise suppression) with the ByteTrack tracking algorithm to enable stable extraction of vehicle trajectories under complex occlusion conditions. Finally, by combining functional zone division within the stand, a semantic map is constructed, and a behavior analysis method based on a spatiotemporal finite state machine is proposed. This method performs joint reasoning by fusing multi-dimensional constraints including position, zone, and time, enabling automatic detection of abnormal behaviors such as “intrusion into restricted areas” and “abnormal stop.” Quantitative evaluations demonstrate the framework’s efficacy: it achieves an average physical localization error (RMSE) of 0.32 m, and the improved detection model reaches an accuracy (mAP@50) of 90.4% for ground support vehicles. In tests simulating typical violation scenarios, the system achieved high recall (96.0%) and precision (95.8%) rates in detecting ‘area intrusion’ and ‘abnormal stop’ violations, respectively. These results, achieved using only existing surveillance cameras, validate its potential as a cost-effective and easily deployable tool to augment existing safety monitoring systems for airport ground operations.

## 1. Introduction

The continuous expansion of the civil aviation industry has led to exponential growth in the number of global flight takeoffs and landings, significantly increasing the operational pressure on airport ground support systems. As the critical physical interface connecting airborne flights and ground support operations, the aircraft stand is one of the most operationally dense and interactively complex areas within the air transport network. In this confined space, aircraft, various types of ground support equipment (GSE), and operational personnel interact dynamically and frequently, resulting in a high concentration of safety risks. According to statistics from the International Air Transport Association (IATA) and related insurance institutions, the global annual direct and indirect economic losses caused by improper GSE operations (e.g., scraping, collisions, etc.) amount to as high as USD 10 billion [1]. Therefore, achieving comprehensive, all-weather, and fine-grained monitoring of the aircraft stand has become a core task in advancing the development of “smart airports” and enhancing the safety management capabilities of ground operations.

Currently, the operational supervision of aircraft stands at large hub airports primarily relies on collaborative sensing technologies centered around surface movement radar (SMR) and multilateration systems (MLATs) [2]. Although research indicates that enhancing the automation level of ground handling operations is crucial for reducing accident risks [3], the mainstream solution widely adopted in the industry remains the Advanced Surface Movement Guidance and Control System (A-SMGCS), developed based on standards established by the European Organization for the Safety of Air Navigation (Eurocontrol) [4]. This system aims to address visual blind spots in air traffic control towers and systematically manage ground operational risks by integrating multi-source surveillance data. However, significant limitations persist in the “last-mile” phase of ground operations at aircraft stands. Firstly, the dense structural environment within stand areas—including jet bridges, high-mast lights, and aircraft fuselages—often obstructs SMR signals, leading to multipath effects and detection blind spots that compromise positioning continuity [5,6]. Secondly, as a cooperative surveillance technology, MLAT operates by relying on signals transmitted by onboard transponders of targets. However, due to cost and operational constraints, a considerable number of ground support vehicles—such as baggage tractors, water service trucks, and specialized operational vehicles—are not equipped with transponders or ADS-B transmitters. As a result, these non-cooperative targets remain “invisible” within the existing surveillance framework, creating notable gaps in safety oversight [7].

Computer vision technology, leveraging its comprehensive advantages in deployment flexibility, system cost, information dimensionality, and compatibility with existing infrastructure, is widely regarded as a crucial technical pathway to address the surveillance blind spots of traditional radar systems [8]. However, achieving reliable and intelligent monitoring of aircraft stand environments directly using existing monocular surveillance cameras at airports still faces several key technical challenges:

1. Lack of physical spatial measurement: Monocular surveillance images exhibit significant perspective distortion. Observations in the pixel coordinate system cannot be directly converted into physical spatial distance and scale information, making it difficult to accurately determine whether vehicles have intruded into safety-restricted zones with stringent physical dimension constraints, such as engine intake hazard areas [9,10].
2. Complex occlusions and scene interference: In the dynamic aircraft stand environment, support vehicles frequently pass beneath aircraft wings or are occluded by fixed structures like jet bridges, resulting in partial or complete loss of target appearance features. Meanwhile, background noise such as oil stains on the ground and complex marking lines significantly interferes with the feature extraction and robustness of lightweight detection models [11].
3. Insufficient high-level behavioral semantic modeling: Existing research methods predominantly focus on low-level visual tasks such as object detection and tracking. They lack the capability for structured modeling and interpretable reasoning of complex spatiotemporal behaviors with clear safety semantics, such as “abnormal stop” and “area intrusion,” making it difficult to support intelligent regulatory decision-making [12].

To address the above challenges, this paper proposes a lightweight vision-based framework for vehicle state perception and spatiotemporal behavior analysis oriented towards aircraft stand safety. The main contributions of this framework are structured into three progressively refined core layers:

Preprocessing Layer: By utilizing the inherent linear features of the scene, monocular camera self-calibration and physical plane mapping are achieved. This establishes an accurate transformation model to map image pixel coordinates to actual physical coordinates, effectively resolving the lack of a physical measurement baseline in monocular vision.

Perception Layer: An improved lightweight YOLO detector is paired with the ByteTrack algorithm for robust vehicle detection and continuous trajectory tracking, even under complex occlusion conditions. Specifically, structural improvements were made to the YOLO network by incorporating Ghost modules to reduce computational redundancy and Convolutional Block Attention Modules (CBAMs) to enhance target feature extraction against complex background noise.

Semantic Decision Layer: By combining a dynamic semantic map with a spatiotemporal finite state machine (FSM), the physical trajectories of vehicles are transformed into structured behavioral descriptions. This categorizes vehicle behaviors to automatically detect and judge typical safety events such as “normal driving,” “area intrusion,” and “abnormal stop”.

Compared to traditional high-cost surface movement radar systems, the proposed method relies solely on existing monocular surveillance cameras at airports, requiring no additional hardware installation or facility modifications. While significantly reducing costs, it offers superior engineering feasibility and potential for broader adoption.

## 2. Related Work

### 2.1. Video Surveillance for Aircraft Stands and Ground Support Operation Analysis

With the rapid advancement of computer vision technology, video analytics has become a crucial technical means for extending civil aviation intelligent transportation systems into the domain of ground operations. Early research primarily focused on enhancing operational efficiency and surveillance performance. For instance, Yıldız et al. extracted key operational timestamps by detecting the movements of ground support vehicles to optimize maintenance processes [12]. Other studies have attempted to analyze motion patterns using machine learning methods to improve situational awareness in air traffic control systems [13]. In the context of automated operations, Zhang et al. proposed a vision-based navigation framework for unmanned ground support vehicles [14], while Ruiz-Barroso et al. explored real-time unsupervised object localization methods based on edge computing [15]. However, existing research has largely concentrated on timestamp extraction or single-vehicle behavior control, lacking a systemic analysis and supervision of the complex interactions among multiple targets within aircraft stand areas from a global situational awareness perspective. Consequently, these approaches struggle to effectively address the operational safety risks posed by a large number of non-cooperative targets.

### 2.2. Monocular Camera Calibration and Scene Geometric Correction

Establishing an accurate mapping relationship between images and physical space is a prerequisite for monitoring functions such as intrusion detection and speed measurement. In the field of traffic surveillance, using parallel lane lines to extract vanishing points for camera self-calibration has become a classical method [16]. By introducing the Transformer architecture, Li et al. further enhanced the adaptability and robustness of this method under complex road conditions [17]. To address the calibration needs of large-scale surveillance networks, Guo et al. and Rameau et al. proposed an online self-calibration framework and a universal global calibration toolbox, respectively [18,19]. Additionally, the research by Zhou et al. on geospatial registration and Rosten et al. on wide-angle lens distortion correction has laid an important theoretical foundation for related technologies [20,21].

Although aircraft stand scenarios lack long continuous lane lines, their strictly standardized guidance markings and functional area boundaries align with the geometric assumptions of the “Manhattan world,” making them suitable for calibration methods based on linear features. By extracting these structured visual features, high-precision homography transformation can be achieved, thereby establishing a stable mapping from the image coordinate system to the Aircraft Stand physical coordinate system.

### 2.3. Lightweight Object Detection and Multi-Object Tracking

Aircraft stand edge monitoring tasks impose stringent requirements on the real-time performance and perceptual accuracy of algorithms. The YOLO series of algorithms, owing to their favorable balance between speed and accuracy, have been widely adopted as detection frameworks in this domain [22]. To accommodate the resource-constrained deployment environment of edge devices, researchers have successively proposed lightweight improvement schemes such as GhostNet [23], the work by Xing et al. [24], and Ghost-YOLO-GBH [25]. By introducing strategies such as linear transformations, attention mechanisms, and multi-scale feature fusion, these approaches significantly reduce the computational overhead of models. Although the latest versions, such as YOLOv9 and v10, along with their customized variants for traffic scenarios, continue to push the performance boundaries [26,27,28], their deployment efficiency on edge devices still requires further optimization.

In terms of object tracking, to address the insufficient association capability of DeepSORT and SORT algorithms under complex occlusion conditions [29], ByteTrack proposed by Zhang et al. effectively mitigates tracking loss caused by occlusion and background interference by fully utilizing low-confidence detection boxes for trajectory association [30]. Furthermore, improvements in nonlinear motion modeling and appearance feature utilization by algorithms such as OC-SORT and BoT-SORT [31,32] provide new technical insights for robust tracking of densely interacting multiple targets in aircraft stand scenario.

### 2.4. Trajectory Analysis and Anomalous Behavior Recognition

Methods for assessing behavioral compliance can be broadly categorized into data-driven and rule-driven approaches. Data-driven methods typically employ networks such as LSTM or CNN to learn the spatiotemporal distribution patterns of normal trajectories, thereby enabling anomaly detection [33,34,35,36]. While such methods possess the capability to uncover potential unknown risks, their decision-making processes lack interpretability, making it difficult to meet the stringent requirements of aviation safety regulation for event attribution and accountability.

In contrast, rule-based methods hold greater practical value in strongly regulated scenarios such as aircraft stand operation safety. For instance, the ROAM framework proposed by Li et al. [37] and the sequence analysis methods based on finite state machines (FSMs) by Arslan et al. [38] and Zhang et al. [39] all model behavioral logic through explicit rules. Given that stand operations are governed by strict standards such as AHM913 [40], FSM methods combined with semantic maps can map vehicle trajectories to region states with clear physical meanings, thereby generating interpretable violation alerts. Furthermore, research by Li et al. on semantic-aware subtrajectory detection [41] provides important theoretical support for constructing behavior analysis frameworks with spatiotemporal interpretability.

To systematically summarize the aforementioned literature and explicitly highlight the research gaps addressed in this study, Table 1 presents a comparison of the existing methods, their key contributions, and their limitations in the context of aircraft stand safety supervision.

## 3. Problem Formulation and Overall System Framework

To address the core challenges in monitoring non-cooperative targets at aircraft stands—namely, the lack of visual localization, unstable dynamic tracking, and complex semantic judgment of violations—this study constructs a hierarchical and progressively refined lightweight intelligent visual perception system for aircraft stands. The system achieves real-time conversion from raw video streams to interpretable safety events based on a monocular camera.

To achieve reliable and comprehensive surveillance in a real-world aircraft stand, managing the extreme complexity of dynamic operations is paramount. A typical turnaround process involves multiple ground support vehicles, passenger stairs, and personnel operating simultaneously. In such highly congested environments, a “worst-case scenario” frequently occurs: targets are completely occluded by the large aircraft fuselage or heavy equipment for extended periods.

**Figure 1:**
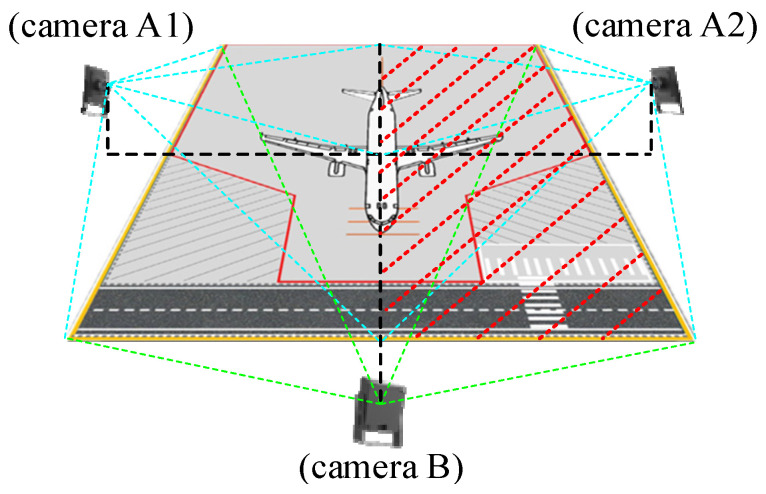
Conceptual diagram of a multi-camera deployment strategy for comprehensive aircraft stand surveillance. Cameras A1 and A2 (blue) monitor the lateral sides, while Camera B (green) provides a frontal view to overcome worst-case occlusions from wings and passenger stairs.

To fundamentally address these visual blind spots, a complete airport deployment requires a multi-camera cooperative network, as conceptualized in Figure 1. In this envisioned topology, Cameras A1 and A2 are deployed to monitor the port and starboard sides of the aircraft, respectively. However, relying solely on side views inevitably leads to severe occlusions caused by aircraft wings and passenger stairs. Therefore, introducing a frontal/nose-view camera (Camera B) is critical. The overlapping field of view from Camera B captures targets hidden from the A1/A2 perspectives, significantly mitigating detection instability and identity loss caused by prolonged occlusions, thereby enhancing the overall robustness of the surveillance system.

**Figure 2:**
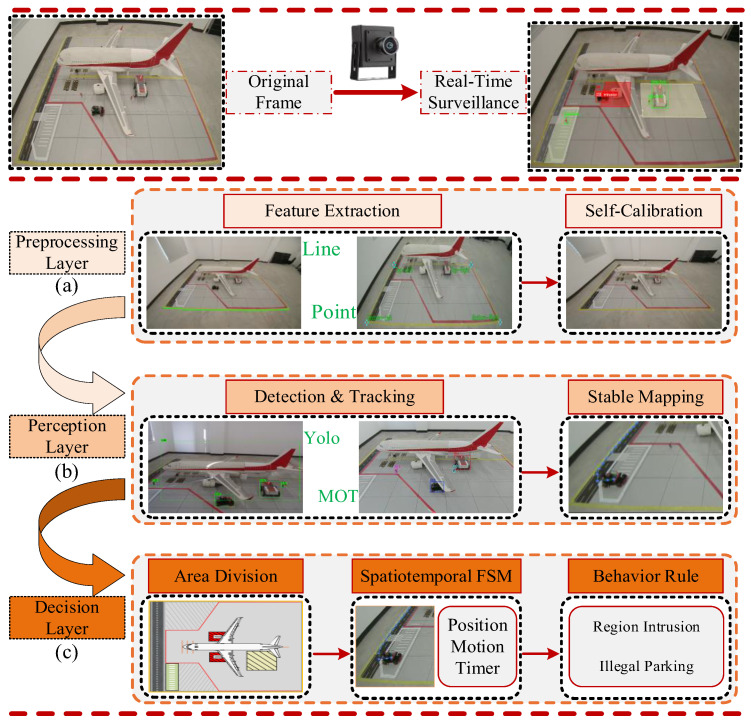
Overall System Architecture Diagram. (**a**) Preprocessing Layer: Establishes the physical plane mapping by extracting scene-constrained linear and point features to solve the homography transformation. (**b**) Perception Layer: Integrates the improved lightweight YOLO detector and ByteTrack algorithm to achieve robust vehicle tracking and stable localization in the physical coordinate system. (**c**) Decision Layer: Defines functional semantic zones and utilizes a spatiotemporal Finite State Machine (FSM) to perform joint reasoning for safety event recognition, such as area intrusion and abnormal stop.

While a multi-sensor fusion network represents the ultimate solution for airport deployment, achieving reliable multi-camera spatial registration and cross-view trajectory association first requires a highly robust and accurate single-node visual perception foundation. Therefore, this current study explicitly narrows its scope to focus on establishing a high-precision algorithmic framework for a single side-view surveillance node (representing the A2 perspective). The proposed monocular system aims to solve the core challenges of physical planar mapping, lightweight target perception, and interpretable behavioral reasoning, laying a crucial technical groundwork for future multi-camera fusion research. The overall single-node system architecture is illustrated in Figure 2.

The algorithmic framework comprises three fundamental layers: the Preprocessing Layer, the Perception Layer, and the Decision Layer.

In the Preprocessing Layer, the system primarily accomplishes the initialization of environmental parameters and the establishment of spatial reference. First, the feature extraction module analyzes input video frames, automatically detecting and extracting key structural features with stable geometric significance from the scene (such as lines and corners) using image processing techniques. Subsequently, the self-calibration module solves for camera intrinsic parameters, distortion coefficients, and extrinsic matrices based on the geometric constraints formed by these features. It establishes an accurate mapping relationship from the image pixel coordinate system to the physical coordinate system of the aircraft stand via homography transformation, providing a unified measurement reference for subsequent perception and decision-making.

The Perception Layer is responsible for real-time detection and state estimation of vehicle targets. The detection and tracking module employs the improved lightweight YOLO model and multi-object tracking algorithm developed in this study to achieve bounding box detection and cross-frame identity association of vehicles in complex stand environments. The stable mapping module receives pixel-level trajectory data, converts it into position coordinates in the physical coordinate system in real time using the mapping matrix generated in the preprocessing layer, and suppresses detection noise through temporal filtering methods. This ultimately outputs smooth, continuous vehicle motion trajectories with practical physical significance.

The Semantic Decision Layer, located at the top of the system architecture, is tasked with converting the physical states of vehicles into interpretable safety event outputs. This layer first defines functionally semantic electronic fences (e.g., restricted areas, driving zones, etc.) in the physical coordinate system through the region division module, in accordance with aircraft stand operational specifications. The spatiotemporal state machine maintains the operational context of each vehicle in real time, dynamically tracking its position, motion state, and dwell time. The behavioral rule module performs multi-dimensional reasoning based on preset logical rules: it executes real-time spatial judgment for “area intrusion” behavior and conducts joint analysis combining spatiotemporal information for “abnormal stop” behavior, ultimately generating corresponding visualized violation alert information.

## 4. Scene-Constrained Geometric Correction and Planar Mapping

After establishing the overall system framework, the primary challenge lies in eliminating geometric distortions in the monocular vision system. To address this, this chapter systematically elaborates on the proposed hierarchical geometric correction method.

**Figure 3:**
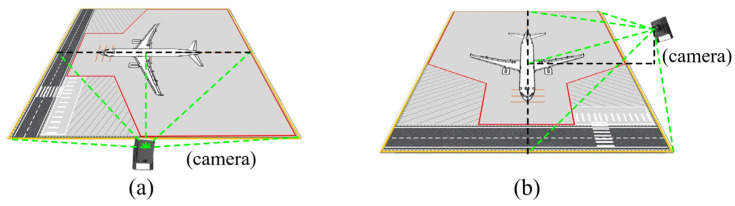
Camera–Aircraft Stand Relationship Diagram: (**a**) Side view; (**b**) Front view.

In the context of monocular visual surveillance for aircraft stands, radial distortion introduced by wide-angle lenses and perspective deformation resulting from high-angle oblique viewing cause image pixel coordinates to fail to directly correspond to actual distances and shapes in physical space. To achieve accurate mapping from “raw video frames” to the “physical plane of the aircraft stand,” this chapter proposes a hierarchical geometric correction pipeline: First, by extracting long linear features with significant geometric constraints from the scene, self-calibration of camera intrinsic parameters and distortion coefficients is accomplished. Subsequently, key corner points are further located in the distortion-corrected images, and a homography matrix is computed based on these feature points, thereby establishing a stable mapping relationship between the image pixel coordinate system and the physical coordinate system of the aircraft stand. The spatial geometric relationship between the camera and the aircraft stand is illustrated in Figure 3.

### 4.1. Morphology-Based Scene Linear Feature Extraction

**Figure 4:**
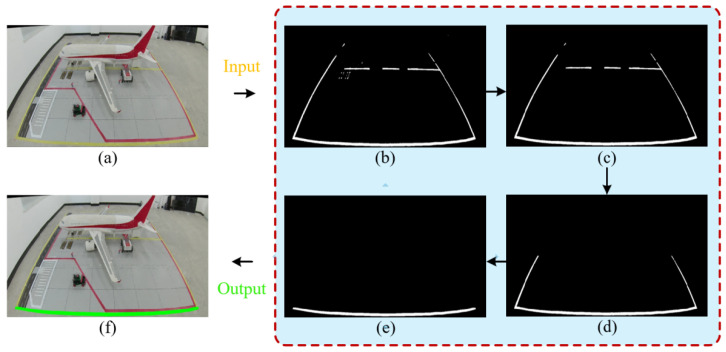
Flowchart of the linear feature extraction algorithm: (**a**) Original input; (**b**) Color segmentation result; (**c**) Noise suppression result; (**d**) ROI focusing result; (**e**) Morphological processing result; (**f**) Linear feature extraction result.

Linear structures present in the scene provide natural geometric constraints for correcting camera radial distortion. However, due to factors such as varying lighting conditions and wear on ground markings, traditional edge detection methods often struggle to extract continuous and complete linear features. To address this, this study proposes a linear feature extraction strategy based on “Color Enhancement and Morphological Synergy,” the detailed algorithmic flow of which is illustrated in Figure 4.

(1) Color Feature Extraction: Aims to separate the yellow marking targets in the aircraft stand scene from the complex background. By leveraging the illumination robustness of the HSV color space, target regions are segmented by setting hue thresholds. The preliminary mask after color segmentation is shown in Figure 4b.
(2) Noise Suppression and ROI Focus: To eliminate interference from ground oil stains and background clutter, connected component analysis is first applied to filter out small noise points with areas below a set threshold, as shown in Figure 4c. Subsequently, based on prior geometric knowledge of the aircraft stand scene, a region of interest (ROI) is defined to exclude interference from distant stand backgrounds and dynamic vehicles, retaining only the lower half of the image containing critical markings, as illustrated in Figure 4d.
(3) Anisotropic Morphological Optimization: To address the geometric characteristics of ground markings—”laterally extended and longitudinally narrow”—a cascaded morphological filter is designed. First, a vertical small-kernel opening operation is applied to remove vertical interferences such as vehicle shadows. Then, a large-scale horizontal closing operation is employed to robustly repair line breaks caused by reflections or occlusions, ensuring feature connectivity. The result after morphological processing is shown in Figure 4e.
(4) Geometric Line Extraction: Building on the morphological processing, a column-wise scanning algorithm is applied to traverse the binary mask. By extracting the bottom edge points of the effective pixel regions in each column, wide line features are transformed into precise discrete point sets. Finally, geometric lines describing the aircraft stand boundaries are fitted, as shown in Figure 4f.

### 4.2. Camera Self-Calibration Based on Line Constraints

**Figure 5:**
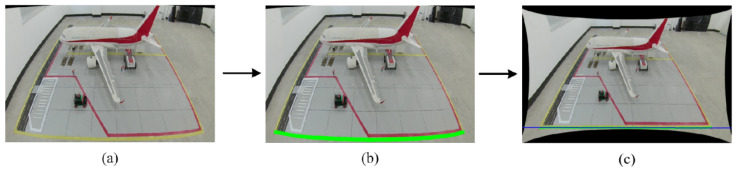
Schematic of the self-calibration algorithm: (**a**) Original image; (**b**) Image after extracting linear features; (**c**) Image after self-calibration based on linear features.

The radial distortion of wide-angle lenses causes straight lines in physical space to appear curved in images. Based on the curved point sets extracted in Section 4.1, this section utilizes the principle of line constraints to inversely derive distortion parameters and restore geometric linearity in the images, as illustrated in Figure 5. This study employs a two-parameter radial distortion model to describe the relationship between the original distorted points $\left(x_{d},y_{d}\right)$ and the corrected ideal points $\left(x_{u},y_{u}\right)$, as shown in Equations (1)–(3).

(1) $$x_{u}=x_{d}\left(1+k_{1}r^{2}+k_{2}r^{4}\right)$$

(2) $$y_{u}=y_{d}\left(1+k_{1}r^{2}+k_{2}r^{4}\right)$$

(3) $$r^{2}={x_{d}}^{2}+{y_{d}}^{2}$$

where $r^{2}$ is the squared distance from the point to the image center, and $k_{1},k_{2}$ are the radial distortion coefficients to be optimized.

To solve for the optimal radial distortion coefficients $k_{1\;}and\;k_{2}$, this paper constructs an objective function based on the collinearity constraints of straight lines. Let $P_{line}=\{L_{1},L_{2},\ldots ,L_{n}\}$ represent the set of *n* feature lines extracted from the scene, where each line $L_{i}$ consists of a set of pixel coordinate points $\left\{p_{i,1},p_{i,2},\ldots ,p_{i,m}\right\}$.

The objective function is defined as the sum of distances from the corrected points to the fitted line $l_{i}$, as shown in Equation (4).

(4) $$f(k_{1},k_{2})=\min\sum_{i=1}^{n}\sum_{j=1}^{m}dist\left(g(p_{i,j};k_{1},k_{2}),l_{i}\right)$$

where g(⋅) is the radial distortion correction mapping function. By minimizing this objective function, curved edge features in the image are restored to straight lines in physical space.

The algorithm constructs a nonlinear optimization objective function, which aims to solve for the optimal parameter combination $\left(k_{1},k_{2}\right)$ to minimize the geometric–topological deviation between the corrected point set and the ideal straight line. This ensures that the new point set $P_{line}$, derived from correcting the original point set ${P^{\prime }}_{line}$, is geometrically and topologically closest to a straight line. By minimizing the fitting residuals, the optimal distortion parameters are solved. Subsequently, the original video frames undergo geometric resampling to generate distortion-corrected images. A schematic of the algorithm is illustrated in Figure 5 below.

### 4.3. Feature Point Extraction and Planar Mapping

**Figure 6:**
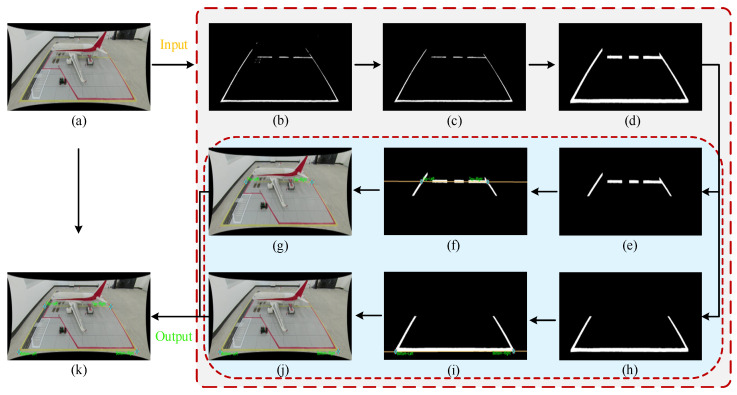
Flowchart of the point feature extraction algorithm: (**a**) Original input image; (**b**) HSV color space threshold segmentation result; (**c**) Denoised binary mask; (**d**) Vertical morphological dilation result; (**e**) Far-end region ROI extraction; (**f**) Upper line fitting and intersection computation; (**g**) Far-end feature point mapping; (**h**) Near-end region ROI extraction; (**i**) Near-end line fitting and endpoint search; (**j**) Near-end feature point mapping; (**k**) Extracted four feature points.

After distortion correction, the image restores linear features consistent with perspective projection. To convert pixel coordinates to physical coordinates, a homographic mapping between the two planes must be established. However, in actual aircraft stand scenarios, the far-end corner points of the stand’s rectangular area are often difficult to detect accurately due to perspective foreshortening. To address this, this study proposes a feature point localization method based on “Virtual Reconstruction”, with the process illustrated in Figure 6.

The feature extraction strategy leverages the geometric priors of the corrected image. It first inputs the distortion-corrected image and then uses HSV color space segmentation to extract ground marking features. Due to the thinning and blurring of far-end lines caused by perspective effects, a vertical morphological dilation operation is applied to enhance the saliency and connectivity of the target area, providing a foundation for subsequent fitting. To address the invisibility of far-end corner points, the algorithm crops the upper ROI region of the image and employs the RANSAC algorithm for robust line fitting on the remaining discrete edge points. By calculating the intersection points between the fitted virtual line and the left and right boundary lines of the aircraft stand, the missing coordinates of the far-end corner points are reconstructed under strong occlusion conditions. Simultaneously, in the lower ROI region of the image, an edge scanning method is used to locate the leftmost and rightmost physical endpoints of the near-field lines. Finally, the spatial fusion of the far-end virtual intersection points and the near-end measured endpoints establishes the four feature points.

Obtaining these four high-confidence feature points indicates that the corresponding constraints between pixel space and physical space have been established. Combining the prior knowledge of the standard physical geometric dimensions of the aircraft stand, the extracted image plane feature point coordinates (x,y) are paired with their corresponding physical world coordinates $\left(X_{w},Y_{w}\right)$. Assuming the local aircraft stand ground approximates a flat 2D plane and the ground markings generally adhere to orthogonal geometric constraints (aligning with the Manhattan-world assumption), the projective transformation relationship between them can be described by a 3 × 3 homography matrix H, with the transformation relationship expressed as Equation (5).

(5) $$s\left[\begin{array}{c} x\\ y\\ 1 \end{array}\right]=H\left[\begin{array}{c} X_{w}\\ Y_{w}\\ 1 \end{array}\right]=\left[\begin{array}{ccc} h_{11} & h_{12} & h_{13}\\ h_{21} & h_{22} & h_{23}\\ h_{31} & h_{32} & h_{33} \end{array}\right]\left[\begin{array}{c} X_{w}\\ Y_{w}\\ 1 \end{array}\right]$$

where s is the scale factor for homogeneous coordinate normalization, and H is the homography matrix describing the planar projective transformation relationship.

**Figure 7:**
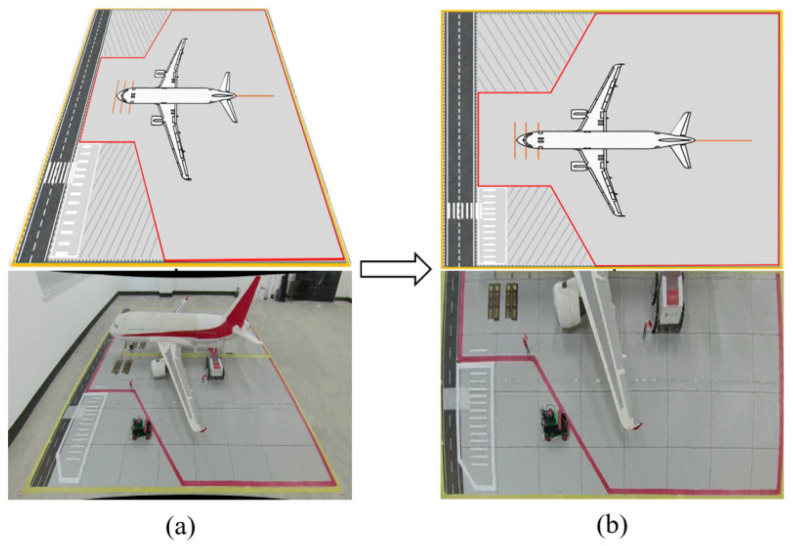
Schematic diagram of planar mapping: (**a**) Original pixel plane; (**b**) Mapped physical plane.

Using the aforementioned four pairs of corresponding points, the matrix H can be solved via the Direct Linear Transformation (DLT) algorithm. Once this mapping relationship is established, any target pixel point detected in the image can be mapped back to the physical plane through matrix inversion. With this mapping model, the system achieves accurate conversion from “pixel space” to “metric physical space,” providing a unified and precise spatial reference for subsequent vehicle trajectory analysis and speed calculation. The effect of the planar mapping is illustrated in Figure 7.

### 4.4. Assumptions and Robustness Analysis of the Method

The self-calibration and homography mapping method proposed in this study is based on two core geometric assumptions:

1. ”Manhattan World” Assumption: The aircraft stand ground is assumed to be a planar surface, and the ground markings (such as lead-in lines and stand boundary lines) are assumed to be mutually parallel or perpendicular in physical space. This provides the necessary geometric constraints to solve for distortion parameters and establish mapping using linear features.
2. Constant Camera Intrinsics Assumption: It is assumed that internal parameters, including focal length and principal points, remain constant during the monitoring process.

As indicated in Table 2, conditions in real-world operational environments may deviate from idealizations. To evaluate the robustness of the framework, targeted evaluations were conducted:

Deviation from the “Manhattan World” Assumption: Extreme camera tilt or localized wear of ground markings can degrade the accuracy of linear feature extraction. Factors such as extreme perspective angles and varying ambient illumination (e.g., low-light or low-contrast conditions) are primary considerations regarding the method’s robustness. The sensitivity of the calibration process to these environmental variables is evaluated through comparative experiments in the subsequent Results section.

Deviation from the “Flat Plane” Assumption: In practice, aircraft stand surfaces may not be absolutely flat due to drainage requirements or structural variations. The actual apron ground may have slight slopes, but the frame can tolerate such geometric errors. The experimental section conducted a quantitative analysis of positioning errors under non-ideal conditions.

In summary, the proposed method demonstrates favorable robustness under typical aircraft stand monitoring scenarios. While its performance may decrease under extreme perspectives or severe weather conditions, these limitations represent directions for future optimization through multi-camera fusion or the introduction of dynamic models.

## 5. Object Detection and Tracking

### 5.1. Improved YOLO-Based Object Detection Algorithm

In aircraft stand operational scenarios, visual perception of Ground Support Equipment (GSE) faces dual challenges: limited computational resources and severe background interference. To meet the real-time inference requirements of embedded edge devices, detection models must exhibit extremely low parameter counts and computational latency. Simultaneously, factors such as complex ground markings, oil stains, and dynamic shadows cast by aircraft can easily interfere with feature extraction, leading to missed detections of small or partially occluded objects.

**Figure 8:**
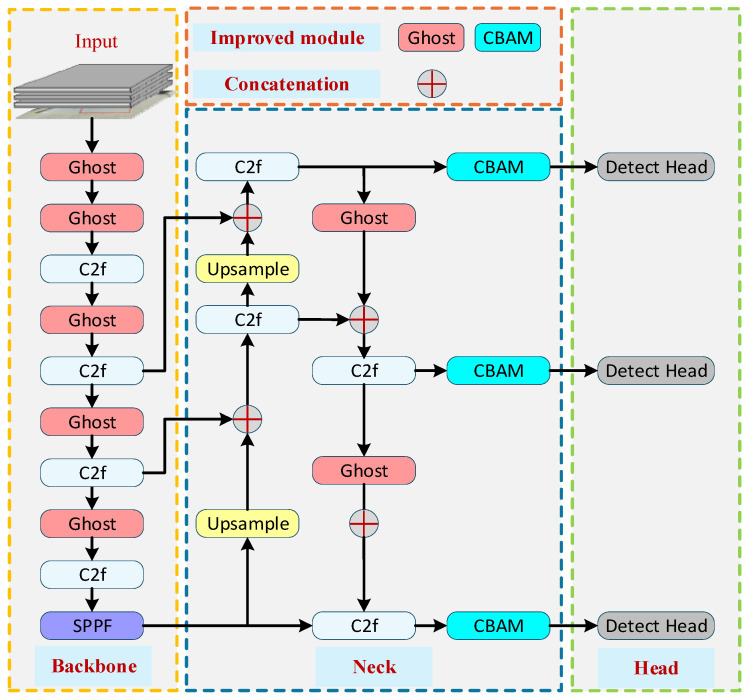
Architecture Diagram of the Improved YOLOv8n Model.

Although the baseline YOLOv8n model offers a favorable balance between speed and accuracy, its standard convolutional operations exhibit computational redundancies and lack an attention mechanism tailored to these complex environments. To address these issues, this study performs targeted lightweight and feature enhancement improvements on the YOLOv8n model, as illustrated in Figure 8. The proposed modifications are characterized by three core aspects:

- Lightweight Network Reconstruction: Ghost modules replace standard convolutional units to generate “ghost feature maps” via lightweight linear transformations, significantly reducing parameters and FLOPs while maintaining representation capability.
- Attention-Driven Feature Enhancement: A Convolutional Block Attention Module (CBAM) is integrated into the Neck network to adaptively calibrate features in both channel and spatial dimensions, suppressing background noise and focusing on vehicle targets.
- Training Strategy Optimization: To ensure robust convergence and enhance model generalization in complex aircraft stand environments, a composite training strategy is implemented. This includes intensive online data augmentation to mitigate sample imbalance for small-scale targets. Furthermore, the training process is governed by an SGD optimizer paired with a Cosine Annealing learning rate scheduling strategy. (details in Section 7.1.2).

**Figure 9:**
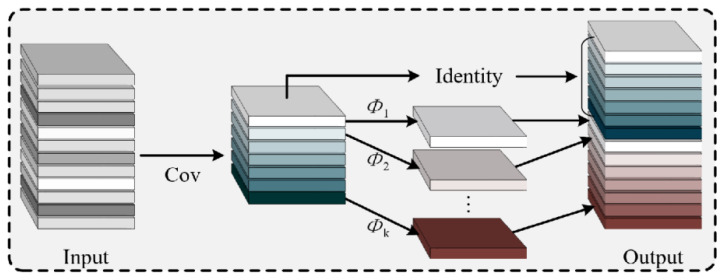
Schematic diagram of the Ghost module.

Given the characteristics of aircraft stand surveillance video streams—large data volumes and the need for real-time response—the model must first address the inefficiency of traditional convolution operations. In deep convolutional neural networks, feature maps often exhibit significant redundancy. While this redundancy aids in comprehensively representing visual features, it also incurs substantial computational costs. To mitigate this, this study employs Ghost modules to replace some of the standard convolutional units in the backbone network. These modules adopt a “divide-and-conquer” design philosophy: first, a small number of convolutional kernels generate a set of “intrinsic feature maps” containing primary structural information; subsequently, lightweight linear transformation operations are applied to these intrinsic features to generate complementary “ghost feature maps.” This mechanism significantly reduces both parameter count and floating-point operations (FLOPs) while effectively preserving feature representation capability. The working principle of this approach is illustrated in Figure 9. By incorporating Ghost modules, the improved network significantly reduces memory usage and inference latency, thereby better adapting to deployment environments on airport edge devices.

**Figure 10:**
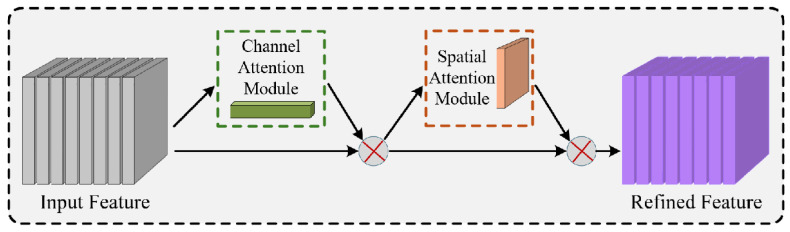
Schematic diagram of the CBAM module.

On the basis of achieving model lightweighting, to further address the misdetection issues caused by “highly structured” environments and “low visual contrast” in aircraft stand scenarios, the algorithm requires more precise feature screening capabilities. Due to the similarity in color between operational vehicles and concrete ground surfaces and the interference caused by complex ground markings in extracting vehicle contours, this study introduces the Convolutional Block Attention Module (CBAM) at key positions in the feature fusion network (Neck). As shown in Figure 10, this module consists of two cascaded sub-modules: channel attention and spatial attention. These sub-modules adaptively calibrate the feature maps from the channel and spatial dimensions, respectively, thereby suppressing irrelevant background responses and enhancing feature activation in target regions.

Specifically, the channel attention module models interdependencies among channels, assigning higher weights to feature channels that contain critical discriminative information about vehicles, while suppressing responses from channels primarily conveying background information such as ground textures. The spatial attention module compresses feature maps along the spatial dimension and generates an attention heatmap, guiding the model to focus on target regions amidst complex background interference. Particularly for low-contrast situations, such as when ground oil stains and dynamic shadows share similar color profiles with operational vehicles, the CBAM module, through its spatial attention mechanism, effectively suppresses responses in these non-target areas. This targeted calibration guides the model to focus strictly on the contour and texture features of the vehicles themselves, thereby significantly improving detection robustness under challenging visual conditions.

### 5.2. Multi-Object Tracking

After completing vehicle object detection, a multi-object tracking algorithm (MOT) is required to associate detection boxes across different frames into continuous trajectory sequences. In aircraft stand scenarios, vehicles often pass beneath aircraft wings or experience brief occlusion by other operational vehicles, leading to detection box loss or reduced confidence. Traditional tracking methods typically discard low-confidence detection boxes directly, resulting in frequent trajectory interruptions, which subsequently affect the accurate accumulation and determination of dwell time in behavioral analyses such as “abnormal stop.”

To address this, this study introduces the ByteTrack tracking algorithm, whose core advantage lies in its unique “dual matching” mechanism: it not only associates high-confidence detection boxes but also effectively utilizes potential target information from low-confidence detection boxes through a secondary matching strategy. The specific tracking process is as follows:

#### 5.2.1. Initial Matching

Based on Kalman filtering, the positions of existing trajectories in the current frame are predicted, and high-confidence detection boxes are prioritized for IoU matching with the predicted trajectories.

#### 5.2.2. Secondary Matching and Mining

For trajectories that remain unassociated after the initial matching, a secondary matching is performed within the set of low-confidence detection boxes. This step effectively recovers targets whose detection scores have dropped due to occlusion, motion blur, or other factors, significantly enhancing trajectory continuity and completeness.

#### 5.2.3. Trajectory State Update

For successfully matched targets, their Kalman filter states are updated using the current detection boxes. If a trajectory fails to be successfully matched over consecutive frames, the target is considered to have left the monitoring area, and the corresponding trajectory ID is terminated.

### 5.3. Real-Time Stable Localization in Physical Space

The vehicle trajectories obtained from the aforementioned detection and tracking modules only contain positional information (x,y) in the image pixel coordinate system, which cannot be directly used to determine whether a vehicle has entered “absolute restricted areas” or “electronic fences” defined by strict physical dimensions. To construct physical trajectories with metric units (meters) and practical measurement significance, this section employs a spatial mapping strategy to convert detection results from the pixel domain into aircraft stand physical coordinates in real time.

Specifically, the geometric center point of the detection box is selected as the representative feature point for the vehicle’s position. Using the homography matrix H obtained through scene self-calibration in Section 4, the tracked pixel center point $P_{img}(x,y)$ of the vehicle in each frame is mapped in real time to the aircraft stand physical coordinate system $\left(X_{w},Y_{w}\right)$ via the inverse transformation of Equation (6).

(6) $$\left[\begin{array}{c} X_{w}\\ Y_{w}\\ 1 \end{array}\right]=s^{\prime }H^{-1}\left[\begin{array}{c} x\\ y\\ 1 \end{array}\right]$$

where $X_{w},Y_{w}$ denote the lateral and longitudinal coordinates of the vehicle in the aircraft stand physical plane coordinate system; $H^{-1}$ represents the inverse of the homography matrix H derived in Section 4; and x,y represent the pixel coordinates of the detection box center in the image coordinate system.

**Figure 11:**
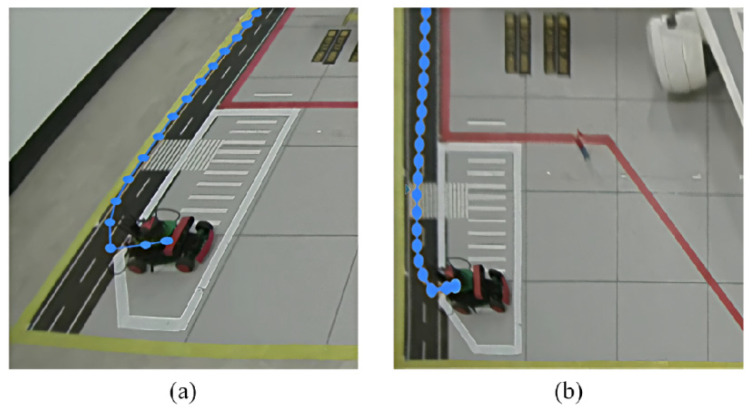
Schematic comparison between pixel-domain trajectories and physical-plane trajectories: (**a**) Raw trajectories in the pixel coordinate system; (**b**) Corresponding trajectories transformed onto the physical plane.

Through this mapping, the nonlinear pixel displacements affected by perspective distortion in the image are transformed into linear straight-line distances on the physical plane. Combined with the stable identity IDs maintained by the ByteTrack algorithm, the system can directly output vehicle trajectory sequences containing physical location information: $Trk_{w}=\{P_{w1},P_{w2},\ldots ,P_{wt}\}$. These unfiltered raw physical coordinates preserve the most authentic state of the vehicle’s motion and will be directly used as input data for the subsequent “spatiotemporal state machine.” This data is utilized to calculate the vehicle’s instantaneous speed and determine its spatial inclusion relationship with polygonal electronic fences, thereby achieving a transition from “pixel perception” to “physical cognition”, as illustrated in Figure 11.

## 6. Spatiotemporal Semantic-Based Aircraft Stand Vehicle Behavior Analysis

In the previous chapter, continuous motion trajectories of vehicles in the aircraft stand physical coordinate system, denoted as $Trk=\{P_{1},P_{2},\ldots ,P_{t}\}$, were successfully obtained through geometric projection and temporal smoothing. To achieve automated monitoring of aircraft stand safety conditions, this chapter proposes a behavior analysis method based on “semantic regions + temporal states.” The method first constructs a polygonal semantic map according to the functional planning of the stand, utilizing geometric topological relationships to determine the spatial attributes of vehicles. Subsequently, a spatiotemporal state machine is established to enable joint reasoning over vehicle position, motion state, and dwell time, thereby achieving automated detection of typical violation behaviors such as area intrusion and abnormal stop.

### 6.1. Semantic Region Construction for Aircraft Stand Scenarios

**Figure 12:**
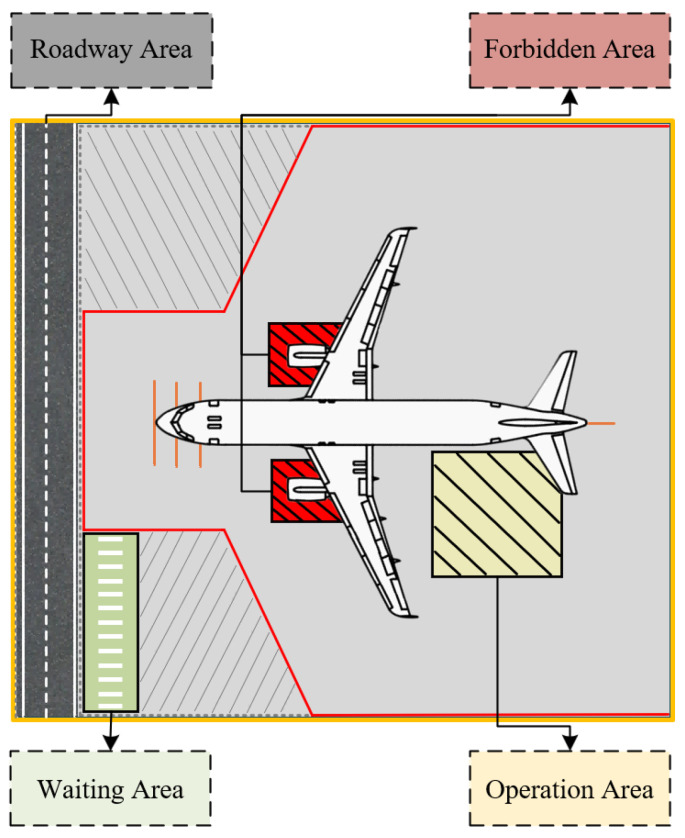
Schematic diagram of semantic region partitioning and electronic fencing for aircraft stand.

To establish the correlation between vehicle physical coordinates and safety supervision rules, it is first necessary to construct a semantic map of the aircraft stand scenario. Based on the “Technical Standards for Flight Areas of Civil Airports” issued by the Civil Aviation Administration of China and combined with the actual distribution of ground markings at the stand [42], this study divides the physical plane of the aircraft stand into four types of regions with clear topological constraints and functional definitions, forming a digital “electronic fence,” as illustrated in Figure 12.

#### 6.1.1. Absolute Restricted Area (Forbidden Area)

This refers to high-risk zones strictly prohibited for any vehicle entry, mainly including aircraft engine intake hazard areas and wingtip clearance protection zones, denoted as $R_{forbid}$.

#### 6.1.2. Roadway Area

This refers to designated vehicle-only travel lanes within the aircraft stand, denoted as $R_{road}$. According to the operating rules, vehicles are only allowed to pass through this area, and any form of long-term stay is prohibited.

#### 6.1.3. Operation Restricted Area

This refers to temporary operational zones delineated around aircraft support interfaces (such as cargo doors and refueling panels), denoted as $R_{work}$. This area allows support vehicles to park and perform operations within specified timeframes, subject to relevant operational duration and safety distance restrictions.

#### 6.1.4. Waiting Area

This refers to designated zones around the aircraft stand for support vehicles to safely wait and temporarily park before receiving operational instructions, denoted as $R_{wait}$. Vehicles in this area must remain stationary until granted permission to enter the operation restricted area or proceed to the roadway.

Mathematically, each of the aforementioned regions $R_{k}$ is defined as a closed, simple two-dimensional polygon, described by an ordered set of vertices $V_{k}=\{v_{1},v_{2},\ldots ,v_{n}\}$ on the physical plane. For any given time t, determining the spatial relationship between the position coordinates $P_{t}^{i}=(X_{t},Y_{t})$ of vehicle *i* on the physical plane and the semantic regions translates into the classic geometric problem of point-in-polygon testing.

The spatial state function $\Omega (P_{t})$ is defined as shown in Equation (7).

(7) $$\Omega (P_{t})=\left\{\begin{array}{ll} State_{forbid}, & if\;P_{t}\in R_{forbid}\\ State_{road}, & if\;P_{t}\in R_{road}\\ State_{work}, & if\;P_{t}\in R_{work}\\ State_{wait}, & if\;P_{t}\in R_{wait}\\ State_{other}, & otherwise \end{array}\right.$$

### 6.2. Definition of Vehicle Spatiotemporal State Machine

Relying solely on spatial position cannot distinguish between “normal driving” and “abnormal stop.” To address this, a vehicle state vector incorporating motion characteristics and time dimensions is constructed. The state $S_{t}^{i}$ of vehicle i at time t is defined as a quadruple, as shown in Equation (8).

(8) $$S_{t}^{i}=\langle Pos_{t},Zone_{t},Motion_{t},Timer_{t}\rangle$$

where $Pos_{t}$ represents the smoothed physical coordinates $\left(X_{t},Y_{t}\right)$ in the current frame; $Zone_{t}$ denotes the type of semantic region currently occupied, i.e., the output of $\Omega (Pos_{t})$; $Motion_{t}$ indicates the binarized motion state, with possible values {Static,Moving}; and $Timer_{t}$ represents a duration counter for a specific state.

Motion Status Determination: Considering the minor jitter present in monocular visual localization, directly determining instantaneous zero velocity is prone to misjudgment. This study adopts a short-term displacement threshold method. If the cumulative displacement of a vehicle within a sliding time window $\Delta T_{win}$ is less than a preset threshold $\delta_{static}$, it is classified as static; otherwise, it is classified as moving, as shown in Equation (9).

(9) $$Motion_{t}=\left\{\begin{array}{ll} Static, & if\;\sum_{k=t-\Delta T_{win}}^{t}\|P_{k}-P_{k-1}{\|}_{2}<\delta_{static}\\ Moving, & otherwise \end{array}\right.$$

### 6.3. Anomalous Behavior Inference Rules

Based on the above definitions, complex aircraft stand violations are modeled as a set of deterministic spatiotemporal logic rules.

Rule 1: Area Intrusion Detection

Area intrusion is an “instant-trigger” event that does not depend on time accumulation. As long as a vehicle’s physical coordinates fall within the polygon of a restricted area, the system immediately triggers a high-level alert. The logic for area intrusion determination is shown in Equation (10).

(10) $$\exists t,\;if\;(Zone_{t}==State_{forbid})$$

Rule 2: Abnormal Stop Detection

Abnormal Stop is a “spatiotemporal composite” event. The detection logic is defined as follows: a vehicle is located within a roadway area and remains in a “static” state for a duration exceeding the permissible maximum threshold $T_{limit}$.

The logic for abnormal stop determination is shown in Equation (11).

(11) $$if\;(Zone_{t}==State_{road})\wedge (Motion_{t}==Static)\wedge (Timer_{t}>T_{limit})$$

## 7. Experiments and Analysis

This chapter aims to validate the performance and applicability of the proposed lightweight visual perception and spatiotemporal behavior analysis framework in real aircraft stand operational scenarios. The experimental design follows a progressive logic of “physical mapping → object perception → behavior understanding”: first, a reproducible sandbox-based aircraft stand perception dataset and test platform are constructed; second, a multi-dimensional evaluation system covering physical mapping accuracy, object detection and tracking performance, and behavior recognition accuracy is established; finally, through hierarchical experiments, core modules such as scene feature extraction, camera self-calibration, object detection, trajectory association, and violation behavior judgment are quantitatively evaluated.

### 7.1. Experimental Environment and Dataset

#### 7.1.1. Experimental Platform and Parameter Configuration

To simulate the actual working conditions of airport edge computing nodes and ensure the reproducibility of the experiments, all tests were conducted on a workstation running the Windows 11 operating system. The hardware platform configuration includes an Intel Core i9-13900K processor, 64 GB of system memory, and an NVIDIA RTX A4000 GPU with 16 GB of dedicated video memory. The software environment is built on Python 3.11.13, utilizing the PyTorch 2.9.1 deep learning framework, with GPU acceleration enabled via CUDA 12.6.

The key hyperparameter settings for model training in all experiments are as follows:

- Input image size: 640 × 640;
- Epoch: 200;
- Initial learning rate: 0.01;
- Weight decay: 0.05;
- Batch size: 8;
- Optimizer: SGD;
- Patience: 20.

#### 7.1.2. Dataset

Due to the lack of aircraft-stand-specific categories in publicly available datasets and the difficulty in capturing low-probability anomaly scenarios, such as unauthorized intrusions, this study independently constructed a dedicated dataset tailored for aircraft stand perception. Data collection was conducted on a 1:10 scaled physical simulation platform, which serves as more than a geometric replication; it is meticulously designed to reproduce the core visual challenges of real-world aircraft stand surveillance.

Regarding distortion characteristics, the platform incorporates lenses with radial distortion effects to authentically simulate the wide-angle optical properties of airport cameras, ensuring the self-calibration algorithm can effectively handle nonlinear deformations. To validate illumination robustness, the experiments encompass a variety of complex lighting conditions—including high exposure, low illumination, and partial shadow occlusion—confirming the adaptability of the HSV-based preprocessing module to significant brightness variations. Furthermore, the platform utilizes scaled aircraft and vehicle models to recreate physical occlusion effects, such as targets passing under wings or being obstructed by the fuselage and jet bridges, providing a rigorous environment for tracking and behavior analysis.

Crucially, all reported physical spatial metrics have been scaled by a factor of 10 to accurately reflect meter-level measurement errors in real-world scenarios. For instance, a measured distance of 0.1 m on the platform corresponds to 1 m in actual space, and the reported localization RMSE of 0.32 m represents the estimated error within the real-world coordinate system.

**Figure 13:**
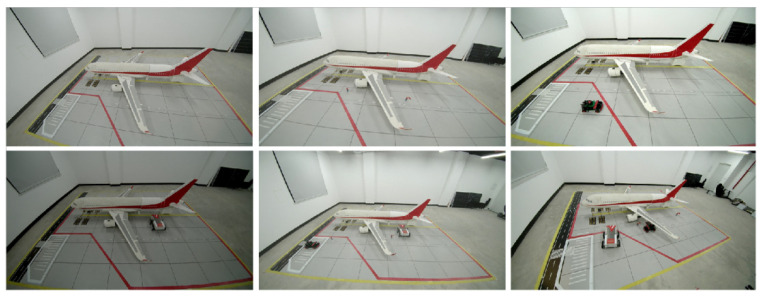
Dedicated dataset for aircraft stand perception.

The dataset comprises 800 carefully selected images, encompassing variations in lighting intensity, shooting perspectives, and occlusion scenarios, as exemplified in Figure 13. The annotated objects are categorized into four core classes: Airplane, Galley_Truck, Ground_Crew, and GSE (introduced as unmanned equipment). To ensure objective evaluation, the dataset is divided into training and validation sets in an 8:2 ratio. During the training phase, multiple online data augmentation strategies were employed, including Mosaic splicing, MixUp blending, and HSV color space perturbation, to enhance the model’s adaptability to small targets and complex lighting conditions.

#### 7.1.3. Complexity Analysis of Experimental Scenarios and Deployment Considerations

To bridge the gap between simulation and real-world application, a phased validation roadmap has been established. The first phase, presented in this study, focuses on verifying the geometric and perceptual feasibility of the core algorithms within a 1:10 scaled physical simulation platform. While this platform authentically reproduces wide-angle lens optical distortion and physical occlusion effects, we acknowledge that achieving reliable and comprehensive surveillance of an aircraft stand requires addressing the extreme complexity of dynamic operations. As conceptualized in Figure 1, a complete airport deployment necessitates a multi-camera cooperative network where Cameras A1 and A2 monitor the port and starboard sides, respectively. However, since side views inevitably suffer from severe occlusions caused by aircraft wings and equipment, the introduction of a frontal nose-view camera (Camera B) is critical to capture targets hidden from the A1/A2 perspectives and mitigate identity loss during prolonged occlusions.

Although a multi-sensor fusion network represents the ultimate solution, the robust and accurate performance of a single-node visual perception foundation is the prerequisite for multi-camera spatial registration and cross-view trajectory association. Therefore, this study explicitly focuses on establishing high-precision algorithms for a single side-view surveillance node (representing the A2 perspective) to solve the core challenges of physical mapping and behavioral reasoning. Building upon this foundation, the second phase of research is currently in progress, involving cooperation with airports to collect real-world data from existing fixed surveillance cameras. This phase constructs complex datasets containing more dynamic objects, such as passenger stairs, personnel, and baggage, to specifically validate tracking and behavior recognition under realistic occlusion. Finally, the third phase of future work will focus on deploying multi-camera systems to investigate cross-camera target hand-off and information fusion mechanisms while conducting long-term stability and performance evaluations in real operational aircraft stand environments.

### 7.2. Evaluation Metrics

To comprehensively evaluate the multi-layered framework proposed in this study, the evaluation metrics are carefully selected to align with the specific mathematical nature of each sub-task. For the perception layer, object detection and tracking are evaluated using standard computer vision metrics (such as mAP and MOTA). For physical localization, since it is a continuous spatial regression task, distance-based error metrics (RMSE, MAE, and MaxError) are utilized to accurately quantify physical coordinate deviations in meters. Conversely, in the semantic decision layer, the recognition of safety events is modeled as a discrete binary classification problem. Thus, Recall and Precision are selected as the optimal metrics to evaluate the system’s sensitivity to true anomalies and its robustness against false alarms.

#### 7.2.1. Metrics for Detection Evaluation

To comprehensively assess the performance of the improved lightweight YOLO model, this study adopts the mean Average Precision (mAP) as the primary evaluation metric for detection accuracy. mAP measures the overall accuracy of the model in multi-class object detection. It is obtained by calculating the area under the Precision–Recall curve for each category and averaging the results, effectively reflecting the model’s recognition capability in complex scenarios. The formula is defined as Equation (12).

(12) $$mAP=\frac{1}{C}\sum_{k=1}^{C}\int_{0}^{1}P_{k}(R_{k})dR_{k}$$

where C is the number of categories and P(R) is the precision–recall curve. Additionally, to assess the feasibility and computational efficiency of the model in practical engineering deployment, lightweight metrics are introduced: GFLOPs, Parameters, and Model Size.

#### 7.2.2. Tracking Evaluation Metrics

Considering that the determination of aircraft stand violations highly depends on the continuity of target identities over long sequences, this experiment focuses on three key metrics for evaluation: MOTA, IDSW, and IDF1.

MOTA: This metric comprehensively considers missed detections, false positives, and identity switch errors, providing an intuitive reflection of the overall accuracy of the tracking algorithm in dense scenarios, as shown in Equation (13).

(13) $$MOTA=1-\frac{\sum_{t=1}^{T}(FN_{t}+FP_{t}+IDSW_{t})}{\sum_{t=1}^{T}GT_{t}}$$

where t is the video frame index, $FN_{t}$, $FP_{t}$, and $IDSW_{t}$ represent the numbers of false negatives, false positives, and identity switches in the *t*-th frame, respectively, and $GT_{t}$ denotes the number of ground-truth targets in that frame.

IDSW: This metric counts the total number of times the tracking algorithm incorrectly assigns different identity IDs to the same real target across the video sequence. It serves as the most critical stability indicator in this study, as an extremely low IDSW value is a prerequisite for ensuring that the timing logic for “abnormal stop” is not abnormally interrupted, as shown in Equation (14).

(14) $$IDSW=\sum_{t=1}^{T}\sum_{i\in GT_{t}}I(ID_{t}^{\left(i\right)}\neq ID_{t-1}^{\left(i\right)})$$

where I(⋅) is the indicator function and $ID_{t}^{\left(i\right)}$ represents the tracking ID matched to the i-th ground-truth target in the t-th frame.

IDF1: This metric measures identity consistency at the entire trajectory level. A higher IDF1 score indicates that the algorithm better maintains the uniqueness of target identities, as shown in Equation (15).

(15) $$IDF1=\frac{2\times IDTP}{2\times IDTP+IDFP+IDFN}$$

where IDTP represents the total number of frames in which target IDs are correctly matched across the entire video, and IDFP and IDFN denote the numbers of false positive and false negative frames at the ID level, respectively.

#### 7.2.3. Metrics for Physical Localization Accuracy Evaluation

To comprehensively assess the impact of geometric correction and temporal filtering modules on the accuracy of vehicle physical coordinate calculation, this experiment selects three key metrics: RMSE, MAE, and MaxError. These metrics quantify the system’s localization performance by calculating the deviation between the algorithm-derived vehicle physical coordinates ${\hat{P}}_{i}$ and the ground-truth physical coordinates $P_{i}$.

RMSE: This metric effectively reflects the overall stability and dispersion of the system’s localization, as shown in Equation (16).

(16) $$RMSE=\sqrt{\frac{1}{N}\sum_{i=1}^{N}||{\hat{P}}_{i}-P_{i}|{|}_{2}^{2}}$$

where N is the total number of test samples, and $||\cdot |{|}_{2}$ denotes the Euclidean distance. A smaller RMSE value indicates that the trajectory points on the physical plane are closer to the true path.

MAE: This metric reflects the average level of system localization deviation, providing an intuitive representation of the overall accuracy of coordinate mapping, as shown in Equation (17).

(17) $$MAE=\frac{1}{N}\sum_{i=1}^{N}||{\hat{P}}_{i}-P_{i}|{|}_{2}$$

where the symbols have the same meanings as in Equation (16). This metric more directly reflects the physical magnitude (m) of the localization error.

MaxError: Considering the specific safety requirements of aircraft stands, relying solely on average metrics may mask extreme localization drift. This metric is used to evaluate the system’s performance under the most severe conditions, as shown in Equation (18).

(18) $$Max\_Error=\underset{i\in \{1\ldots N\}}{\max}||{\hat{P}}_{i}-P_{i}|{|}_{2}$$

where max(⋅) denotes taking the maximum value within the set, used to capture the extreme cases of single-point localization deviation.

#### 7.2.4. Behavior Recognition Evaluation Metrics

The two safety events, “area intrusion” and “abnormal stop,” are treated as binary classification problems and evaluated using the following metrics:

Event Recall: The proportion of correctly identified violation events by the system relative to the total number of true violation events, reflecting the system’s safety assurance capability, as shown in Equation (19).

(19) $$Recall_{event}=\frac{TP_{event}}{TP_{event}+FN_{event}}$$

Alarm Precision: The proportion of true violation events among all alarms issued by the system, reflecting the system’s anti-interference capability, as shown in Equation (20).

(20) $$Precision_{event}=\frac{TP_{event}}{TP_{event}+FP_{event}}$$

### 7.3. Scene Feature Extraction and Self-Calibration Experiments

Accurate extraction of scene geometric features is fundamental to constructing “electronic fences” and achieving physical space mapping. To address interference such as strong reflections, oil stains, and marking wear in aircraft stand scenarios, this section adopts a cascaded extraction strategy based on “color–morphology synergy” and incorporates geometric topology constraints to achieve online camera self-calibration. This experiment aims to validate the robustness of the proposed feature extraction pipeline in complex environments and the geometric accuracy achieved by the homography mapping.

#### 7.3.1. Linear Feature Extraction Experiment

To overcome the instability of traditional gradient operators in extracting weak-texture edges under complex lighting conditions, this study utilizes the insensitivity of the HSV color space to brightness variations in the hue and saturation dimensions for region segmentation. Morphological operations are then applied to repair broken or partially worn markings. During the experiment, by analyzing the hue distribution of yellow guiding lines under different lighting conditions, an adaptive threshold interval was determined. The specific HSV filtering parameters are presented in Table 3.

**Figure 14:**
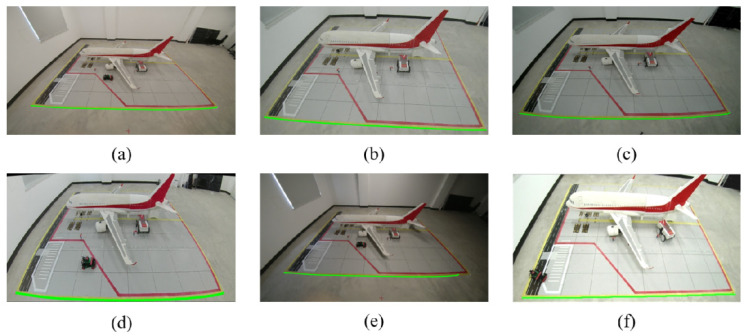
Examples of linear feature extraction in scene characterization. (**a**) Normal lighting conditions; (**b**) Tilted viewing angle conditions; (**c**) Low-illumination dim conditions; (**d**) Different lens distortion conditions; (**e**) Shadow occlusion conditions leading to partial line loss; (**f**) High-exposure conditions.

To systematically simulate the interference of lighting variations and shadows on ground markings in real-world environments, our data collection phase specifically incorporated multiple challenging conditions, including high exposure, low illumination, and partial shadow occlusion (as shown in Figure 14c,e,f). The experimental results indicate that the proposed algorithm demonstrates stable performance across these various typical interference environments. Specifically, the preprocessing based on the HSV color space proves to be highly robust and insensitive to brightness variations. Combined with morphological reconstruction, it effectively restores the topological continuity of the markings, overcoming line breakage issues caused by shadows and ground oil stains to ensure the complete extraction of linear features. Furthermore, in cases of lens distortion and viewing angle tilt, it still accurately captures edge geometric features, comprehensively validating its robustness in complex aircraft stand environments. A comparison of the related extraction effects is shown in Figure 14.

#### 7.3.2. Feature Point Extraction Experiment

**Figure 15:**
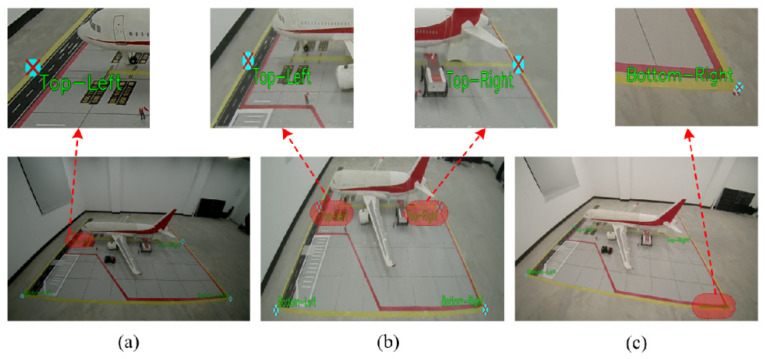
Examples of scene feature point extraction. (**a**) Different lighting conditions; (**b**) Perspective distortion conditions; (**c**) Tilted viewing angle conditions.

To verify the effectiveness of the proposed visual perception system in extracting key points in complex scenes, multi-scenario tests were conducted based on a scaled model of an airport aircraft stand. The experiment focused on evaluating the robustness of the algorithm in feature extraction under different camera perspectives and geometric distortion conditions. The results of feature point extraction in typical scenarios are shown in Figure 15.

The experimental results show that the proposed algorithm possesses strong generalization capability. Under low-light conditions, the algorithm remains unaffected by noise and can accurately detect all key feature corner points. In scenarios with significant perspective distortion or extreme camera tilt angles, the algorithm robustly regresses the coordinates of the four vertices in the marking area by combining edge detection and geometric constraints, without noticeable deviations. This “virtual reconstruction–strict constraint” approach effectively overcomes the failure of traditional corner detection methods in heavily occluded environments, providing reliable feature support for subsequent camera self-calibration.

#### 7.3.3. Scene Self-Calibration & Feature Point Extraction Experiment

**Figure 16:**
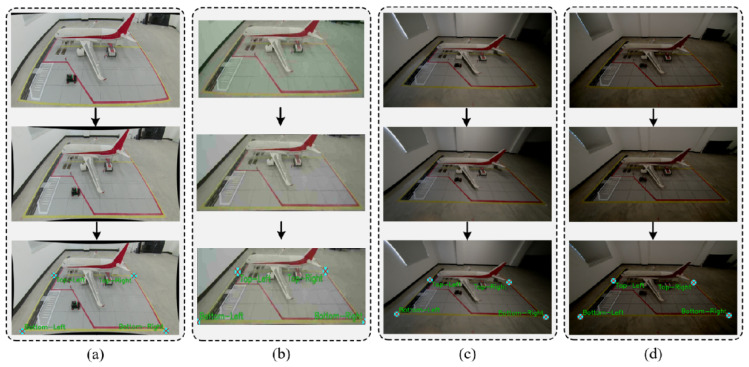
Examples of self-calibration: (**a**) Example 1 with different camera distortions; (**b**) Example 2 with different resolutions; (**c**) Example 3 with insufficient regional lighting; (**d**) Example 4 with varying contrast and low-light conditions.

This experiment aims to evaluate the system’s capability to automatically solve distortion parameters and restore linear geometric characteristics of images based on scene line constraints. The experiment demonstrates the complete processing pipeline from raw video frames to high-precision geometrically corrected images, validating the effectiveness of the “camera self-calibration–feature point visualization” method. Examples of self-calibration processing are shown in Figure 16.

The experimental results indicate that the proposed self-calibration algorithm demonstrates good geometric restoration capability and environmental robustness under various complex working conditions. For different degrees of lens distortion and resolution variations, the algorithm effectively corrects nonlinear deformations, accurately restoring the curved aircraft stand boundary lines and guiding lines affected by wide-angle lenses to strict straight lines, successfully reconstructing the Euclidean geometric structure of the scene. Under conditions of localized insufficient lighting or low-contrast dim light, the algorithm maintains high stability in feature extraction, without significant interference from environmental noise, and the output corrected images clearly retain key texture details.

Finally, the visualization results of superimposed feature points show that the feature points extracted after self-calibration correction are highly consistent with the physical corner point positions. This provides a reliable spatial reference for the subsequent high-precision solving of the homography matrix, effectively overcoming the positioning drift issues often encountered by traditional methods under non-ideal imaging conditions.

#### 7.3.4. Quantitative Reliability Evaluation of Visual Perception Algorithms

To quantitatively assess the reliability of the proposed visual perception system in practical engineering applications, a test dataset comprising 1000 images was constructed, covering five different configurations ranging from ideal to extreme environments. The evaluation metrics are subdivided into three hierarchical and interdependent sub-tasks: Line Extraction, Keypoint Detection, and Self-Calibration. This layered evaluation helps precisely identify the specific stage where algorithm failure occurs. The detailed performance evaluation of each sub-module under different experimental configurations is shown in Table 4.

The experimental data indicate that across the entire test set, the line extraction rate reaches 88.2%, the feature point detection rate is 85.7%, and the final calibration success rate is 83.9%, demonstrating that the system exhibits good effectiveness under most working conditions. Under ideal conditions, all metrics exceed 95%, reflecting high baseline accuracy. Under conditions with angular deviation and lens distortion, the system maintains strong robustness, with calibration success rates of 84.5% and 87.3%, respectively. Although low-light and low-contrast scenarios affect performance to some extent, reducing the calibration success rate to 72.3%, it remains within an acceptable range for engineering applications. Overall, the hierarchical evaluation results verify that the proposed feature extraction and self-calibration algorithms not only effectively restore the geometric structure of the scene but also, in a statistical sense, possess the engineering feasibility to adapt to the complex and variable environments of aircraft stands.

### 7.4. Performance Validation of Object Detection Algorithms

#### 7.4.1. Comparative Experiment with Different Baseline Models

To comprehensively evaluate the performance of the proposed model and verify its comprehensive advantages in aircraft stand edge computing scenarios, this study conducted a full-scale comparison between the improved model and current mainstream lightweight object detection models. The comparison models include the classic YOLOv5 series (v5n, v5s), the widely deployed YOLOv8 series (v8n, v8s), and the newer YOLOv9t and YOLOv10n. To ensure fairness, all models were trained end-to-end on the constructed aircraft stand-specific dataset and evaluated for accuracy, parameter count, and inference speed under the same hardware environment. The detailed performance comparison is shown in Table 5.

The experimental results show that the proposed model achieves a better balance between lightweight design and detection accuracy. As shown in Table 5, the model attains the highest average precision among all compared models: mAP@50 reaches 90.4% and mAP@50:95 reaches 71.2%, significantly outperforming the baseline YOLOv8n and the larger YOLOv8s in terms of performance. At the same time, thanks to the structural design of the Ghost module, the model maintains a highly compact parameter scale, with only 2.15 M parameters and a computational cost of 6.2 GFLOPs, both lower than those of YOLOv8n. This level of efficiency means that the model can be easily deployed on airport edge computing nodes with limited computing power (such as Jetson series devices) to achieve real-time (>30 FPS) video analysis, laying the foundation for its widespread application in practical engineering.

**Figure 17:**
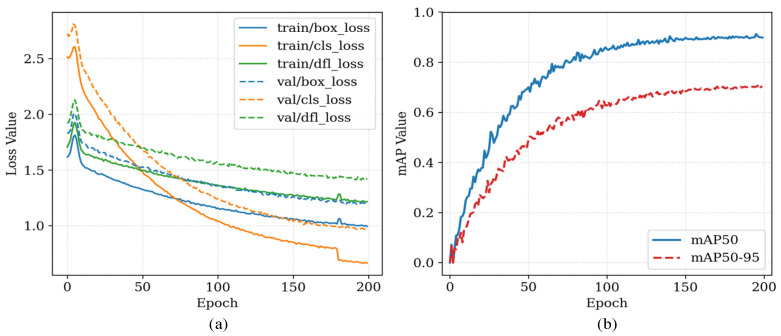
Loss and mAP curves for training and validation. (**a**) Loss curves: showing the changes in box_loss, cls_loss, and dfl_loss of the training and validation sets with epochs; (**b**) mAP curves: showing the changes in mAP50 and mAP50-95 with the number of training epochs.

As shown in Figure 17, although the initial learning rate was set to 0.01, with the help of the SGD optimizer and the cosine annealing learning rate scheduling strategy, the training loss and validation loss of the model decreased steadily and eventually converged. The validation set mAP value also increased and stabilized in sync, without any obvious overfitting or oscillation, which proves the effectiveness of the current hyperparameter settings.

#### 7.4.2. Ablation Study Comparison of Improved Modules

To systematically verify the contribution of the network structure improvements proposed in Section 5.1—namely the introduction of the Ghost modules and the CBAM attention mechanism—we conducted a series of ablation experiments to evaluate their independent effects on the baseline model, as shown in Table 6. The experiment aimed to separately evaluate the impact of the Ghost module and the CBAM attention mechanism on the performance of the baseline model. The introduction of the Ghost module aims to reduce computational redundancy. While its standalone use slightly decreases mAP@50 to 86.1%, it significantly compresses the model, reducing the parameter count from 3.2 M to 1.7 M and the computational cost from 8.7 GFLOPs to 5.0 GFLOPs. In contrast, the CBAM module enhances feature extraction capability by suppressing background noise such as oil stains on the ground, and its standalone introduction increases mAP@50 to 88.3%.

Integrating the two modules generates a synergistic effect through their complementary mechanisms. The fully improved model achieves a 3.9% increase in mAP@50 and a 6.4% increase in mAP@50:95 compared to the baseline. It is noteworthy that these accuracy improvements are accomplished while maintaining practical efficiency; the final model size and computational load still fully meet the requirements for lightweight deployment. These results empirically demonstrate that the lightweight design enabled by the Ghost module and the enhanced feature representation facilitated by the CBAM module can work synergistically, effectively reducing computational overhead while significantly improving object detection accuracy.

**Figure 18:**
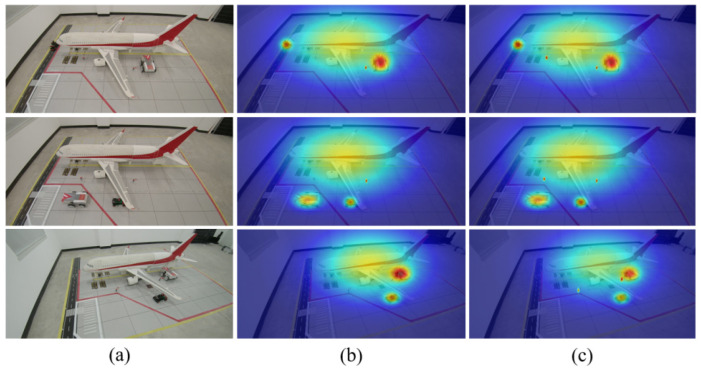
Comparison of heatmaps between the baseline and the improved model. (**a**) Original image; (**b**) Baseline model; (**c**) Improved model.

To further validate the feature extraction capability of the improved model at the visual level, a heatmap visualization comparison was conducted between the baseline model and the improved model, as shown in Figure 18. The observations indicate that the improved model (Figure 18c), under the calibration effect of the CBAM attention mechanism, significantly enhances the feature response to small-scale targets such as Ground_Crew. Even in complex scenarios where vehicles are partially occluded by aircraft wings, it maintains high-confidence activation of the target body without noticeable attention dispersion. This perceptual stability in small object detection and occlusion resistance intuitively explains the reasons behind the model’s significant improvement in detection accuracy under complex aircraft stand environments.

### 7.5. Trajectory Tracking and State Stability Experiment

#### 7.5.1. Evaluation of ByteTrack Tracking Performance

To comprehensively assess the tracking robustness of the ByteTrack algorithm in complex aircraft stand environments, a dedicated tracking test set was constructed. Given the feature diversity of images captured under different camera perspectives and lighting conditions, the experiment directly employed a pre-trained model for object association. To simulate typical challenges in real-world surveillance, multiple highly representative video sequences were selected, covering the most challenging scenarios in aircraft stand environments, including prolonged occlusion of targets by aircraft wings, dense overlapping of multiple vehicles, and significant scale variations as targets move from near to far. All video frames were annotated with bounding boxes and identity IDs using annotation tools. The detailed comparative experimental results are shown in Table 7.

As shown in the quantitative evaluation results in Table 7, ByteTrack demonstrates significant advantages across all key tracking metrics, reflecting its robust performance in typical industrial scenarios. In terms of MOTA, ByteTrack achieves 89.4%, outperforming DeepSORT and OC-SORT by 6.2% and 2.9%, respectively. Notably, in the IDSW metric, which reflects trajectory continuity, ByteTrack records only 11 identity switches, far fewer than DeepSORT’s 63. This indicates that the algorithm can effectively maintain target identity consistency, providing crucial support for time-based cumulative analysis in subsequent behavior judgments such as “abnormal stop.”

**Figure 19:**
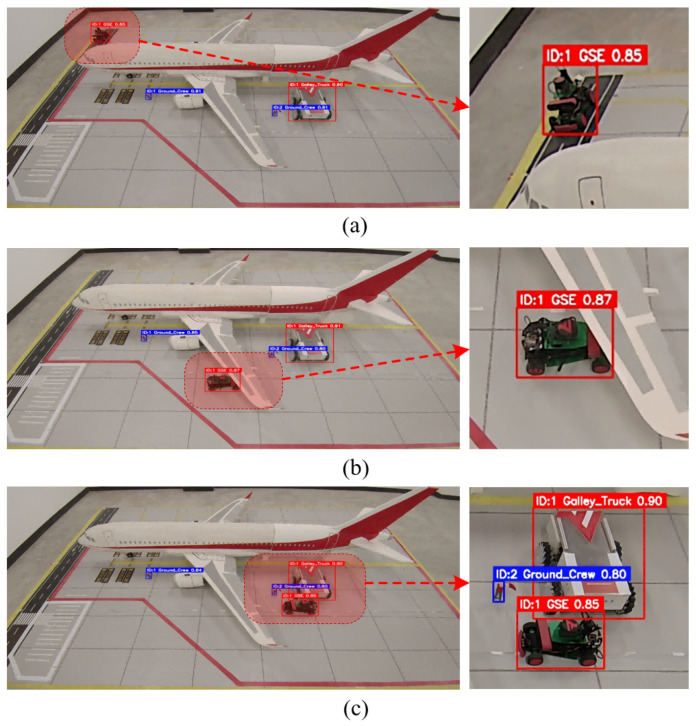
Visualizations of MOT tracking in different scenarios: (**a**) Significant scale variation; (**b**) Partial occlusion by aircraft wing; (**c**) Partial overlap of multiple vehicles in a congested area.

To visually validate the robustness of the tracking algorithm in complex aircraft stand environments, Figure 19 presents a visual comparison of tracking results under three typical challenging scenarios.

#### 7.5.2. Experiment on Physical Trajectory Localization Accuracy

This experiment aims to validate the accuracy of the proposed coordinate mapping model in converting two-dimensional image pixel coordinates to three-dimensional physical space coordinates, with a focus on assessing the system’s ability to recover the true geographical locations of targets in real aircraft stand scenarios. The effectiveness of the planar mapping method is examined by comparing trajectory projection results under two conditions: uncalibrated direct mapping (Uncalibrated) and parameter-optimized calibrated mapping (Calibrated).

**Figure 20:**
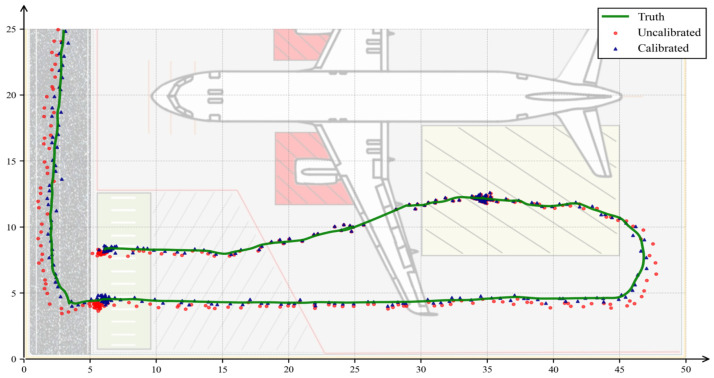
Visualization of Trajectory Mapping (Comparison Before and After Self-Calibration).

The experiment selects a typical operational area of the aircraft stand, records the travel process of target vehicles, and uses positioning equipment to collect trajectories as the ground truth. The pixel center points detected by the visual algorithm are converted to physical coordinates via the mapping model and then compared through visual superposition with the ground truth trajectories. The results are shown in Figure 20.

As shown in Figure 20, the green curve represents the true physical trajectory of the vehicle. The red dots in the figure indicate the trajectory points obtained through direct mapping based on initial parameters. It can be observed that without parameter optimization, the mapping model is significantly affected by perspective distortion, resulting in scale deviations in straight segments and noticeable spatial drift in the lower-right and upper-left regions where distortion is more severe, failing to accurately reflect the actual motion geometry of the vehicle.

In contrast, the planar mapping model after parameter correction effectively overcomes the influence of perspective effects, and the calculated physical coordinate points closely align with the true trajectory. To further quantitatively evaluate the mapping accuracy, the RMSE, Mean Error, and Max Error under both conditions were calculated, with specific data presented in Table 8.

The experimental results indicate that the coordinate mapping model exhibits significant performance differences before and after parameter optimization. After optimization, the root mean square error (RMSE) decreases from 0.5995 m to 0.3204 m, demonstrating that the overall distribution of mapped points is more concentrated and closer to the true trajectory, achieving sub-meter-level localization accuracy. The mean error reduces from 0.522 m to 0.2767 m, indicating effective suppression of systematic bias and more accurate restoration of the target’s physical location. The maximum error also converges from 1.6615 m to 1.0901 m, confirming that outlier errors in distant and edge regions are controlled. These data collectively validate the significant role of parameter optimization in enhancing the geometric mapping accuracy of the model.

In summary, the planar mapping method proposed in this study, combined with the parameter self-calibration mechanism, enables accurate conversion from visual observation data to physical space coordinates. An RMSE of approximately 0.32 m indicates that the mapping model possesses strong spatial geometric recovery capabilities, meeting the practical requirements for position accuracy in aircraft stand electronic fence judgment and vehicle violation behavior analysis.

### 7.6. Violation Behavior Recognition Experiment Based on Spatiotemporal Semantics

#### 7.6.1. Region Intrusion Detection Experiment

**Figure 21:**
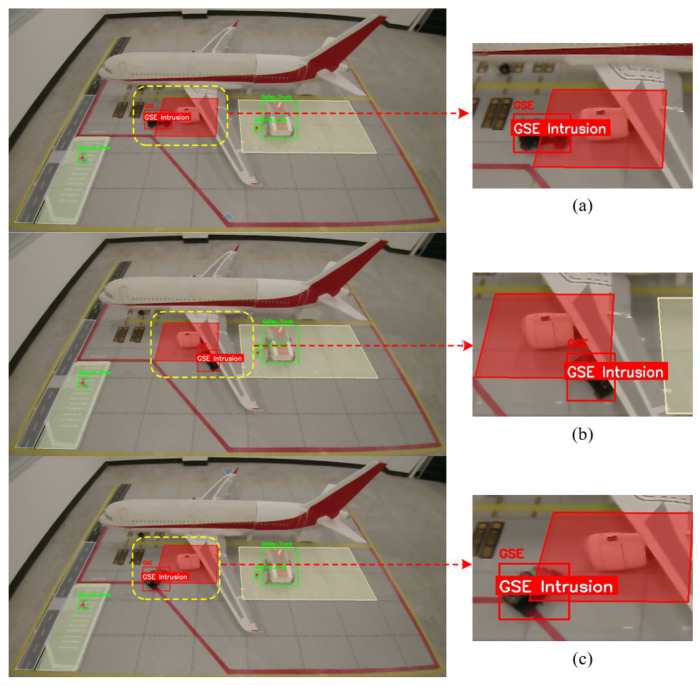
Region Intrusion Detection Diagrams (subfigures (**a**–**c**) visually demonstrate the detection effectiveness of region intrusion from multiple perspectives).

Region intrusion is a high-risk “instant-trigger” event. To thoroughly validate the system’s sensitivity to restricted area boundaries and its stability during prolonged operation, the experiment significantly increased the test sample size. Test vehicles were controlled to cross the predefined red restricted zone (absolute restricted area $R_{forbid}$) a total of 200 times at varying angles (frontal, lateral, tangential) and speeds. The test results are shown in Figure 21.

In the quantitative tests, the experimental results show that out of a total of 200 simulated intrusion tests, the system successfully triggered valid alarms 192 times (TP = 192). The resulting event recall rate (Event Recall) reached 96%. This indicates that the system possesses strong safety assurance capability, effectively capturing instantaneously occurring cross-boundary risks. Additionally, based on efficient homography mapping calculations, the average response delay from vehicle boundary crossing to alarm issuance was less than 200 ms, demonstrating its high reliability in real-time risk control.

#### 7.6.2. Abnormal Stop Detection Experiment

**Figure 22:**
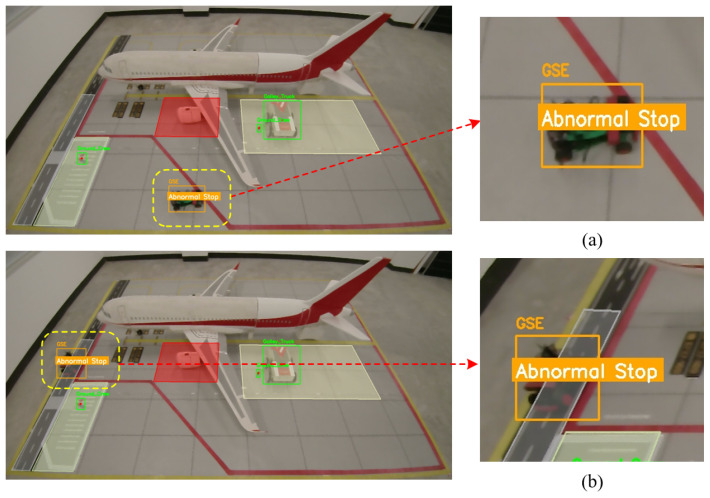
Abnormal Stop Detection Diagrams. (**a**) Abnormal Stop of vehicles in service lanes; (**b**) Abnormal Stop in other areas.

Abnormal Stop is a “spatiotemporal composite” event, and the challenge in its determination lies in distinguishing between “normal yielding (<10 s)” and “Abnormal Stop (>30 s).” To simulate real-world aircraft stand scenarios and ensure the objectivity of the test, this experiment constructed a dedicated test set comprising multiple highly representative video sequences, totaling 3109 frames. The dataset covers complex working conditions such as mixed traffic flow during peak hours, temporary vehicle occlusion by aircraft wings, and lighting variations. The test results are shown in Figure 22.

Test results based on the complete dataset of 3109 frames indicate that the algorithm achieves an Alarm Precision of 95.8% and an Event Recall of 92.0%. The high precision of 95.8% demonstrates the system’s strong anti-interference capability, as the logical reasoning of the spatiotemporal state machine effectively filters out false alarms caused by normal vehicle operation waiting or short-term traffic flow yielding, thereby avoiding unnecessary disturbances to supervisory personnel. Simultaneously, the high recall rate of 92.0% shows that the system can reliably identify the vast majority of abnormal stop behaviors, thereby improving ground operational efficiency while standardizing the traffic order in aircraft stands.

### 7.7. Discussion of Experimental and Analysis Results

This chapter conducts a comprehensive quantitative evaluation of the proposed lightweight visual perception framework across three dimensions: geometric correction accuracy, visual perception performance, and behavioral decision effectiveness, based on a scaled physical simulation environment with realistic geometric constraints and a dedicated test dataset.

First, the experiment validates the robustness of the scene self-calibration module, showing that the system maintains an 83.9% calibration success rate under varying lighting and lens distortion interference, with the root mean square error (RMSE) of physical coordinate mapping as low as 0.32 m. Second, in terms of object detection and tracking, the improved YOLOv8n model achieves an mAP@50 of 90.4% while reducing the parameter count to 2.15 M. Combined with the ByteTrack algorithm, the identity switch count (IDSW) is controlled to 11, effectively mitigating tracking interruptions caused by occlusion at the stand. In behavioral recognition tests, the decision logic based on the spatiotemporal state machine achieves an event recall rate of 96.0% for “area intrusion” scenarios and an alarm precision of 95.8% for “abnormal stop” scenarios. In summary, the hierarchical experimental results indicate that the system possesses engineering capability to handle complex conditions at aircraft stands under monocular visual conditions.

## 8. Conclusions

This study proposes a lightweight vision-based framework for vehicle state perception and spatiotemporal behavior analysis oriented toward aircraft stand safety supervision. The main contributions include (1) a self-calibration and planar mapping technique based on scene line constraints, achieving sub-meter-level physical spatial localization; (2) an improved lightweight YOLOv8n detection model integrated with a ByteTrack tracker, which ensures detection accuracy while effectively mitigating trajectory fragmentation; and (3) a finite state machine (FSM) based on a dynamic semantic map, enabling high-accuracy recognition of area intrusion and abnormal stop behaviors.

Firstly, by employing self-calibration and planar mapping based on scene line constraints, distortions and perspective deformations in wide-angle surveillance images are corrected, achieving a localization accuracy with an RMSE of 0.32 m. Secondly, the YOLOv8n model is enhanced for lightweight and feature augmentation by incorporating Ghost modules and CBAM attention mechanisms, resulting in a 3.9% improvement in mAP@50 compared to the baseline while compressing the model parameters to 2.15 M. Additionally, the ByteTrack tracking algorithm significantly reduces trajectory interruptions caused by vehicle occlusion by effectively associating low-confidence detection boxes, achieving an MOTA score of 89.4%. Finally, the finite state machine constructed on a dynamic semantic map enables precise identification of violations through integrated reasoning of vehicle position, motion state, and dwell time, with recall and precision rates for area intrusion and abnormal stop reaching 96.0% and 95.8%, respectively. Overall experiments demonstrate that the framework exhibits good performance and interpretability in monitoring non-cooperative targets at aircraft stands.

Despite these achievements, this study acknowledges certain limitations: the validation was conducted on a scaled physical platform, which, despite its realistic optical simulation, simplifies the dynamic complexity of real-world aircraft stands involving multiple interacting agents. Furthermore, the reliance on a single camera is inherently insufficient for full stand coverage due to large occlusions by the aircraft itself. Future work will critically focus on (1) investigating multi-camera collaboration and cross-view tracking techniques to overcome the field-of-view limitations and large-object occlusion inherent in single-camera setups and (2) conducting long-term stability validation and performance evaluation of this framework in real operational environments with higher object density and dynamic variability. This research aims to provide a foundational low-cost, vision-based solution that can be scaled and integrated into future multi-sensor airport ground safety surveillance systems.

## Figures and Tables

**Table 1:** Summary of Related Work and Research Gaps.

Research Category	Representative Works	Key Contributions	Research Gaps & Proposed Work Positioning
Aircraft Stand Video Surveillance	Yıldızet et al. [12], Zhang et al. [14]	Extraction of operation timestamps; single-vehicle navigation.	**Gap:** Lacks systematic analysis and supervision of complex interactions of non-cooperative targets within stands. **This work:** Focuses on spatiotemporal behavioral semantic understanding for multi-vehicle targets
Monocular Geometric Correction	You et al. [16], Dubská et al. [9]	Self-calibration utilizing road vanishing points.	**Gap:** Methods rely on long-distance continuous lane lines, unsuitable for aircraft stand scenes with marking breaks and severe occlusion. **This work:** Proposes a feature point localization method based on “color–morphology synergy” and “virtual reconstruction” to adapt to structured aircraft stand environments.
Object Detection & Tracking	Han et al. [23], Zhang et al. [30]	GhostNet lightweight design; ByteTrack tracking algorithm.	**Gap:** Lightweight models lack robustness under strong background interference; tracking algorithms suffer from ID loss under prolonged full occlusion. **This work:** Combines GhostNet with CBAM to enhance anti-interference; verifies ByteTrack’s continuity under aircraft stand-specific occlusion.
Behavior Analysis & Anomaly Detection	Li et al. [37], Arslan et al. [38]	Rule-based/FSM trajectory analysis.	**Gap:** Existing rule-based methods lack precise mapping to physical space; data-driven methods exhibit poor interpretability. **This work:** Proposes a joint reasoning method combining semantic maps and spatiotemporal state machines to transform physical trajectories into interpretable safety events.

**Table 2:** Summary of Core Assumptions and Robustness Analysis.

Core Assumption	Potential Risks (Real-World)	Proposed Mitigation Strategy	Experimental Validation
Ground Planarity	Aircraft stand surfaces may have drainage slopes or structural variations.	Utilization of homography transformation which tolerates localized small-scale unevenness.	Physical localization RMSE remains at 0.32 m even in simulation environments that are not perfectly flat.
Linear Feature Geometry	Ground markings may be worn out or occluded by vehicles.	Synergy of morphological repair and ROI-based “virtual reconstruction”.	The line extraction rate reaches 79.0% even under challenging low-light and low-contrast conditions.
Constant Intrinsics	Auto-focus or auto-iris functions in surveillance cameras may change parameters.	Recommendation of fixing hardware parameters during engineering deployment.	Current success rate is 83.9% overall; future work will introduce online self-calibration.

**Table 3:** HSV Filtering Parameter.

HSV Parameter	Minimum Value	Maximum Value
Hue	20	61
Saturation	74	255
Value	98	255

**Table 4:** Detailed Performance Evaluation of Each Sub-Module Under Different Experimental Configurations.

Experimental Configuration	Total Frames	Line Extraction Rate	Key Point Detection Rate	Self-Calibration Success Rate	Experimental Configuration
Standard View	200	0.972	0.963	0.951	Standard View
Angle Deviation	200	0.885	0.85	0.845	Angle Deviation
Lens Distortion	150	0.913	0.893	0.873	Lens Distortion
Resolution Scale	150	0.933	0.913	0.884	Resolution Scale
Low Light/Contrast	300	0.79	0.756	0.723	Low Light/Contrast
Overall	1000	0.882	0.857	0.839	Overall

**Table 5:** Comparative experiments of different models.

Model	mAP@50 (%)	mAP@50:95 (%)	Parameters (M)	GFLOPs	Model Size (MB)
YOLOv5n	82.1	59.8	1.9	4.5	3.9
YOLOv5s	85.4	63.5	7.2	16.5	14.5
YOLOv8n	86.5	64.8	3.2	8.7	6.2
YOLOv8s	88.9	68.5	11.2	28.6	21.5
YOLOv9t	85.8	64.2	2	7.7	5.8
YOLOv10n	86.2	64.5	2.3	8.2	6.1
Ours	90.4	71.2	2.15	6.2	6.3

**Table 6:** Ablation study results.

Baseline	Ghost	CBAM	mAP50 (%)	mAP50:95 (%)	Parameters (M)	GFLOPs	Model Size (MB)
YOLOv8n			86.5	64.8	3.2	8.7	6.2
	√		86.1	64.2	1.7	5	3.6
		√	88.3	66.9	3.2	8.8	6.3
	√	√	90.4	71.2	2.1	6.2	6.3

**Table 7:** Results of ByteTrack Tracking Performance Evaluation.

Baseline	Method	MOTA (%)	IDF1 (%)	IDSW (Counts)↓
ImprovedYOLOv8n	DeepSORT	83.2	75.8	63
	OC-SORT	86.5	80.4	28
	ByteTrack	89.4	85.1	11

**Table 8:** Physical Trajectory Localization Accuracy Evaluation.

Status	RMSE (m)	Mean Error (m)	Max Error (m)
Uncalibrated	0.5995	0.522	1.6615
Calibrated	0.3204	0.2767	1.0901

## Data Availability

The original contributions presented in this study are included in the article. Further inquiries can be directed to the corresponding author.
